# (*E*)-*N*′-(3,3-Diphenyl­allyl­idene)-*p*-toluene­sulfonohydrazide

**DOI:** 10.1107/S1600536809013269

**Published:** 2009-04-18

**Authors:** Hossein Mehrabi, Reza Kia

**Affiliations:** a Department of Chemistry, Vali-e-Asr University of Rafsanjan, Rafsanjan 77176, Iran; b X-ray Crystallography Unit, School of Physics, Universiti Sains Malaysia, 11800 USM, Penang, Malaysia

## Abstract

In the title compound, C_22_H_20_N_2_O_2_S, the mol­ecule adopts a twisted *E* configuration around the C=N bond. The two phenyl rings are twisted from each other, making a dihedral angle of 78.00 (12)°. The methyl-substituted benzene ring makes dihedral angles of 32.37 (14) and 69.70 (12)° with the two phenyl rings. In the crystal structure, mol­ecules are linked into extended chains along the *b* axis through inter­molecular N—H⋯O hydrogen bonds.

## Related literature

For related compounds and their bioactivities, see; for example, Mehrabi *et al.* (2008 ▶); Tabatabaee *et al.* (2007 ▶); Ali *et al.* (2007 ▶); Tierney *et al.* 2006 ▶; Krygowski *et al.* (1998 ▶); Kayser *et al.* (2004 ▶). For bond-length data, see: Allen *et al.* (1987 ▶).
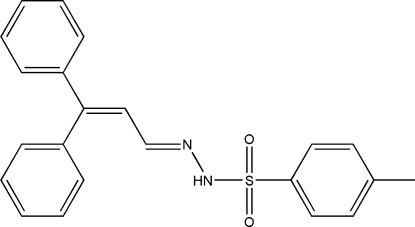


## Experimental

### 

#### Crystal data

C_22_H_20_N_2_O_2_S
*M* *_r_* = 376.46
Monoclinic, 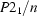

*a* = 14.785 (3) Å
*b* = 6.2179 (12) Å
*c* = 22.519 (5) Å
β = 102.64 (3)°
*V* = 2020.0 (7) Å^3^
*Z* = 4
Mo *K*α radiation
μ = 0.18 mm^−1^
*T* = 294 K
0.50 × 0.28 × 0.12 mm

#### Data collection

Stoe IPDS-II diffractometer
Absorption correction: numerical (*X-RED32*; Stoe & Cie, 2005 ▶) *T*_min_ = 0.940, *T*_max_ = 0.980
5327 measured reflections
5327 independent reflections
4220 reflections with *I* > 2σ(*I*)

#### Refinement

*R*[*F* ^2^ > 2σ(*F* ^2^)] = 0.057
*wR*(*F* ^2^) = 0.156
*S* = 1.10
5327 reflections
249 parameters
H atoms treated by a mixture of independent and constrained refinement
Δρ_max_ = 0.21 e Å^−3^
Δρ_min_ = −0.26 e Å^−3^

### 

Data collection: *X-AREA* (Stoe & Cie, 2005 ▶); cell refinement: *X-AREA*; data reduction: *X-AREA*; program(s) used to solve structure: *SHELXTL* (Sheldrick, 2008 ▶); program(s) used to refine structure: *SHELXTL*; molecular graphics: *SHELXTL*; software used to prepare material for publication: *SHELXTL* and *PLATON* (Spek, 2009 ▶).

## Supplementary Material

Crystal structure: contains datablocks global, I. DOI: 10.1107/S1600536809013269/at2762sup1.cif

Structure factors: contains datablocks I. DOI: 10.1107/S1600536809013269/at2762Isup2.hkl

Additional supplementary materials:  crystallographic information; 3D view; checkCIF report

## Figures and Tables

**Table 1:** Hydrogen-bond geometry (Å, °)

*D*—H⋯*A*	*D*—H	H⋯*A*	*D*⋯*A*	*D*—H⋯*A*
N2—H1N2⋯O2^i^	0.82 (2)	2.11 (2)	2.927 (2)	173 (2)

Symmetry code: (i) 
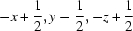
.

**Fig. 1.**
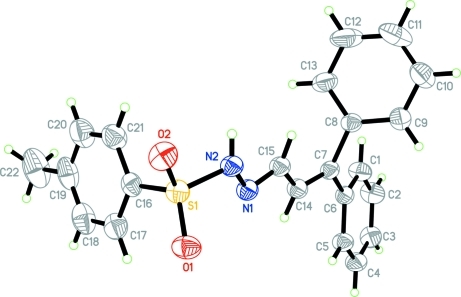
The molecular structure of the title compound, showing 30% probability displacement ellipsoids and the atomic numbering.

**Fig. 2.**
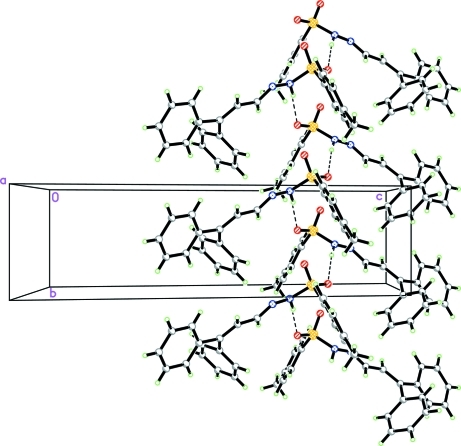
The crystal packing of the title compound, viewed down the a-axis, showing a 1-D extended chain along the b-axis. Intermolecular hydrogen bonds are shown as dashed lines.

## supplementary crystallographic information

### Comment

Sulfonamides were the first class of antimicrobial agents to be discovered. They inhibit dihydropteroate synthetase in the bacterial folic acid pathway. Although their clinical role has diminished, they are still useful in certain situations, because of its efficacy and low cost (Krygowski *et al.*, 1998). Sulfonamides (sulfanilamide, sulfamethoxazole, sulfafurazole) are structural analogs of *p*-aminobenzoic acid (PABA) and compete with PABA to block its conversion to dihydrofolic acid. These agents are generally used in combination with other drugs (usually sulfonamides) to prevent or treat a number of bacterial and parasitic infections (Tierney *et al.*, 2006). Some of the applications of sulfonamides are the anti-infective agents of choice, as follows: Bacteria as Human Pathogens, such as Antibiotic Treatment of Infections Caused by Gram-Positive Bacilli and Gram-negative Haemophilus ducreyi and Haemophilus aegyptius, Alternative Drug for treatment of Chlamydia related diseases (including C. trachomatis, Chlamydia psittaci, Chlamydia pneumonia), Anti-malarial Agents as Dihydropteroate synthetase inhibitors, alternative drugs in tuberculosis treatment, long term treatment of leprosy, treatment of ocular infections. In the latter treatment causative organisms must be identified, and it is preferable to use a drug that is not given systemically. Sulfonamides are also assumed as permitted antibiotics in Pregnancy (Kayser *et al.*, 2004).

In the title compund, (Fig. 1), bond lengths (Allen *et al.*, 1987) and angles are within the normal ranges and are comparable with the related structures (Mehrabi *et al.*, 2008; Ali *et al.* 2007). The molecule adopts a twisted *E* configuration around the C═N bond. The two outer phenyl rings twisted from each other making a dihedral angle of 78.00 (12)°. The methyl-substituted benzene ring makes dihedral angles of 32.37 (14) and 69.70 (12)° with the two outer benzene rings. In the crystal structure the molecule linked together into extended 1-D chains along the *b* axis through intermolecular N—H···O hydrogen bonds (Table 1, Fig. 2).

### Experimental

The synthesis is the same as the earlier report (Mehrabi *et al.*, 2008), except that penylcinnamaldehyde (3 mmol) was used. Single crystals suitable for X-ray analysis were obtained from ethanol solution at room temperature.

### Refinement

H atom bound to N1 was located from a difference Fourier map and refined freely. The rest of the hydrogen atoms were positioned geometrically and refined as riding model with C—H = 0.93–0.96 and *U*_iso_(H) = 1.2 or 1.5 *U*_eq_(C). A rotating group model was used for the methyl group.

### Crystal data

**Table:**

C_22_H_20_N_2_O_2_S	*F*(000) = 792
*M**_r_* = 376.46	*D*_x_ = 1.238 Mg m^−^^3^
Monoclinic, *P*2_1_/*n*	Mo *K*α radiation, λ = 0.71073 Å
Hall symbol: -P 2yn	Cell parameters from 2500 reflections
*a* = 14.785 (3) Å	θ = 2.3–29.2°
*b* = 6.2179 (12) Å	µ = 0.18 mm^−^^1^
*c* = 22.519 (5) Å	*T* = 294 K
β = 102.64 (3)°	Block, colourless
*V* = 2020.0 (7) Å^3^	0.50 × 0.28 × 0.12 mm
*Z* = 4	

### Data collection

**Table:**

Stoe IPDS-II diffractometer	5327 independent reflections
Radiation source: fine-focus sealed tube	4220 reflections with *I* > 2σ(*I*)
graphite	*R*_int_ = 0.0000
Detector resolution: 0.15 pixels mm^-1^	θ_max_ = 29.0°, θ_min_ = 1.9°
rotation method scans	*h* = −20→19
Absorption correction: numerical (*X-RED32*; Stoe & Cie, 2005)	*k* = 0→8
*T*_min_ = 0.940, *T*_max_ = 0.980	*l* = 0→30
5327 measured reflections	

### Refinement

**Table:**

Refinement on *F*^2^	Primary atom site location: structure-invariant direct methods
Least-squares matrix: full	Secondary atom site location: difference Fourier map
*R*[*F*^2^ > 2σ(*F*^2^)] = 0.057	Hydrogen site location: inferred from neighbouring sites
*wR*(*F*^2^) = 0.156	H atoms treated by a mixture of independent and constrained refinement
*S* = 1.10	*w* = 1/[σ^2^(*F*_o_^2^) + (0.0702*P*)^2^ + 0.4245*P*] where *P* = (*F*_o_^2^ + 2*F*_c_^2^)/3
5327 reflections	(Δ/σ)_max_ < 0.001
249 parameters	Δρ_max_ = 0.21 e Å^−^^3^
0 restraints	Δρ_min_ = −0.26 e Å^−^^3^

### Special details

**Table:**

Geometry. All e.s.d.'s (except the e.s.d. in the dihedral angle between two l.s. planes) are estimated using the full covariance matrix. The cell e.s.d.'s are taken into account individually in the estimation of e.s.d.'s in distances, angles and torsion angles; correlations between e.s.d.'s in cell parameters are only used when they are defined by crystal symmetry. An approximate (isotropic) treatment of cell e.s.d.'s is used for estimating e.s.d.'s involving l.s. planes.
Refinement. Refinement of *F*^2^ against ALL reflections. The weighted *R*-factor *wR* and goodness of fit *S* are based on *F*^2^, conventional *R*-factors *R* are based on *F*, with *F* set to zero for negative *F*^2^. The threshold expression of *F*^2^ > σ(*F*^2^) is used only for calculating *R*-factors(gt) *etc*. and is not relevant to the choice of reflections for refinement. *R*-factors based on *F*^2^ are statistically about twice as large as those based on *F*, and *R*- factors based on ALL data will be even larger.

### Fractional atomic coordinates and isotropic or equivalent isotropic displacement parameters (Å^2^)

**Table:**

	*x*	*y*	*z*	*U*_iso_*/*U*_eq_	
S1	0.36602 (3)	0.58739 (7)	0.24598 (2)	0.05559 (15)	
O1	0.39657 (11)	0.7837 (2)	0.22433 (7)	0.0741 (4)	
O2	0.30743 (11)	0.5917 (2)	0.28899 (6)	0.0719 (4)	
N1	0.35283 (10)	0.4163 (2)	0.14209 (6)	0.0542 (3)	
N2	0.30481 (11)	0.4572 (3)	0.18786 (7)	0.0544 (3)	
H1N2	0.2757 (15)	0.355 (4)	0.1974 (10)	0.065 (6)*	
C1	0.35959 (15)	−0.1126 (3)	−0.08028 (10)	0.0681 (5)	
H1	0.3228	−0.2220	−0.0702	0.082*	
C2	0.40308 (17)	−0.1410 (4)	−0.12878 (11)	0.0781 (6)	
H2	0.3953	−0.2693	−0.1505	0.094*	
C3	0.45712 (15)	0.0186 (4)	−0.14457 (9)	0.0722 (6)	
H3	0.4856	−0.0004	−0.1772	0.087*	
C4	0.46904 (15)	0.2069 (4)	−0.11195 (9)	0.0695 (5)	
H4	0.5062	0.3152	−0.1223	0.083*	
C5	0.42599 (14)	0.2365 (3)	−0.06367 (9)	0.0616 (4)	
H5	0.4344	0.3652	−0.0421	0.074*	
C6	0.37052 (12)	0.0769 (3)	−0.04690 (7)	0.0521 (4)	
C7	0.32394 (12)	0.1032 (3)	0.00479 (7)	0.0512 (4)	
C8	0.23773 (13)	−0.0247 (3)	0.00290 (8)	0.0544 (4)	
C9	0.15991 (16)	0.0142 (4)	−0.04264 (10)	0.0781 (6)	
H9	0.1624	0.1197	−0.0715	0.094*	
C10	0.07956 (19)	−0.0983 (5)	−0.04624 (15)	0.0994 (9)	
H10	0.0282	−0.0704	−0.0774	0.119*	
C11	0.0753 (2)	−0.2508 (5)	−0.0041 (2)	0.1177 (12)	
H11	0.0205	−0.3268	−0.0061	0.141*	
C12	0.1509 (3)	−0.2946 (5)	0.04175 (19)	0.1156 (11)	
H12	0.1473	−0.4006	0.0703	0.139*	
C13	0.23312 (18)	−0.1804 (4)	0.04555 (13)	0.0834 (7)	
H13	0.2844	−0.2092	0.0766	0.100*	
C14	0.35757 (13)	0.2367 (3)	0.05125 (8)	0.0569 (4)	
H14	0.4129	0.3070	0.0508	0.068*	
C15	0.31479 (13)	0.2803 (3)	0.10200 (8)	0.0538 (4)	
H15	0.2605	0.2104	0.1053	0.065*	
C16	0.46334 (13)	0.4280 (3)	0.27515 (8)	0.0570 (4)	
C17	0.55009 (15)	0.4897 (5)	0.26802 (10)	0.0759 (6)	
H17	0.5581	0.6184	0.2488	0.091*	
C18	0.62509 (18)	0.3562 (6)	0.29011 (12)	0.0933 (8)	
H18	0.6837	0.3981	0.2858	0.112*	
C19	0.6153 (2)	0.1648 (5)	0.31798 (13)	0.0914 (8)	
C22	0.6986 (2)	0.0211 (6)	0.3407 (2)	0.1415 (16)	
H22A	0.7542	0.0967	0.3381	0.212*	
H22B	0.6932	−0.1064	0.3162	0.212*	
H22C	0.7010	−0.0179	0.3823	0.212*	
C20	0.5286 (2)	0.1072 (4)	0.32492 (14)	0.0966 (9)	
H20	0.5210	−0.0213	0.3444	0.116*	
C21	0.45251 (17)	0.2363 (4)	0.30362 (12)	0.0786 (6)	
H21	0.3942	0.1942	0.3084	0.094*	

### Atomic displacement parameters (Å^2^)

**Table:**

	*U*^11^	*U*^22^	*U*^33^	*U*^12^	*U*^13^	*U*^23^
S1	0.0643 (3)	0.0552 (2)	0.0519 (2)	−0.00057 (19)	0.02298 (19)	−0.01132 (18)
O1	0.0889 (10)	0.0543 (7)	0.0837 (9)	−0.0098 (7)	0.0286 (8)	−0.0086 (7)
O2	0.0832 (9)	0.0781 (9)	0.0640 (8)	0.0105 (7)	0.0370 (7)	−0.0117 (7)
N1	0.0601 (8)	0.0606 (8)	0.0454 (7)	−0.0058 (7)	0.0190 (6)	−0.0035 (6)
N2	0.0594 (8)	0.0600 (9)	0.0480 (7)	−0.0072 (7)	0.0209 (6)	−0.0032 (6)
C1	0.0765 (13)	0.0665 (11)	0.0675 (11)	−0.0158 (10)	0.0293 (10)	−0.0145 (9)
C2	0.0904 (15)	0.0796 (14)	0.0729 (12)	−0.0118 (12)	0.0365 (11)	−0.0235 (11)
C3	0.0722 (12)	0.0916 (15)	0.0594 (10)	0.0002 (11)	0.0290 (9)	−0.0038 (11)
C4	0.0714 (12)	0.0818 (14)	0.0608 (10)	−0.0133 (10)	0.0265 (9)	0.0047 (10)
C5	0.0723 (11)	0.0609 (10)	0.0549 (9)	−0.0121 (9)	0.0208 (8)	−0.0040 (8)
C6	0.0554 (9)	0.0571 (9)	0.0451 (8)	−0.0038 (7)	0.0137 (7)	−0.0010 (7)
C7	0.0578 (9)	0.0527 (9)	0.0444 (7)	−0.0030 (7)	0.0141 (7)	0.0019 (7)
C8	0.0608 (10)	0.0531 (9)	0.0523 (8)	−0.0054 (8)	0.0191 (7)	−0.0029 (7)
C9	0.0677 (12)	0.0984 (16)	0.0666 (12)	−0.0119 (12)	0.0111 (10)	0.0101 (12)
C10	0.0685 (14)	0.119 (2)	0.107 (2)	−0.0174 (15)	0.0096 (14)	−0.0027 (18)
C11	0.0759 (18)	0.097 (2)	0.186 (4)	−0.0279 (16)	0.041 (2)	−0.009 (2)
C12	0.117 (2)	0.0794 (18)	0.161 (3)	−0.0208 (17)	0.053 (2)	0.0344 (19)
C13	0.0860 (15)	0.0677 (13)	0.0986 (17)	−0.0073 (12)	0.0246 (13)	0.0253 (12)
C14	0.0610 (10)	0.0638 (10)	0.0482 (8)	−0.0086 (8)	0.0173 (7)	−0.0043 (7)
C15	0.0587 (9)	0.0567 (9)	0.0482 (8)	−0.0067 (8)	0.0164 (7)	−0.0020 (7)
C16	0.0616 (10)	0.0639 (10)	0.0457 (8)	−0.0021 (8)	0.0125 (7)	−0.0168 (8)
C17	0.0684 (12)	0.0996 (16)	0.0632 (11)	−0.0024 (12)	0.0217 (10)	−0.0067 (11)
C18	0.0626 (13)	0.138 (3)	0.0785 (15)	0.0057 (15)	0.0146 (11)	−0.0242 (17)
C19	0.0841 (16)	0.0917 (18)	0.0844 (16)	0.0213 (14)	−0.0124 (13)	−0.0338 (14)
C22	0.106 (2)	0.132 (3)	0.160 (3)	0.047 (2)	−0.027 (2)	−0.040 (3)
C20	0.0978 (19)	0.0686 (14)	0.107 (2)	0.0050 (13)	−0.0122 (16)	−0.0069 (13)
C21	0.0753 (13)	0.0665 (13)	0.0893 (15)	−0.0043 (11)	0.0075 (11)	−0.0036 (11)

### Geometric parameters (Å, °)

**Table:**

S1—O1	1.4240 (15)	C10—C11	1.353 (5)
S1—O2	1.4341 (14)	C10—H10	0.9300
S1—N2	1.6336 (17)	C11—C12	1.372 (5)
S1—C16	1.752 (2)	C11—H11	0.9300
N1—C15	1.274 (2)	C12—C13	1.394 (4)
N1—N2	1.3969 (19)	C12—H12	0.9300
N2—H1N2	0.82 (2)	C13—H13	0.9300
C1—C6	1.388 (3)	C14—C15	1.448 (2)
C1—C2	1.395 (3)	C14—H14	0.9300
C1—H1	0.9300	C15—H15	0.9300
C2—C3	1.369 (3)	C16—C21	1.380 (3)
C2—H2	0.9300	C16—C17	1.381 (3)
C3—C4	1.373 (3)	C17—C18	1.387 (4)
C3—H3	0.9300	C17—H17	0.9300
C4—C5	1.387 (3)	C18—C19	1.368 (4)
C4—H4	0.9300	C18—H18	0.9300
C5—C6	1.391 (2)	C19—C20	1.373 (4)
C5—H5	0.9300	C19—C22	1.516 (4)
C6—C7	1.486 (2)	C22—H22A	0.9600
C7—C14	1.343 (2)	C22—H22B	0.9600
C7—C8	1.495 (2)	C22—H22C	0.9600
C8—C13	1.376 (3)	C20—C21	1.380 (4)
C8—C9	1.385 (3)	C20—H20	0.9300
C9—C10	1.366 (3)	C21—H21	0.9300
C9—H9	0.9300		
			
O1—S1—O2	119.94 (9)	C10—C11—C12	120.8 (3)
O1—S1—N2	108.18 (9)	C10—C11—H11	119.6
O2—S1—N2	103.87 (9)	C12—C11—H11	119.6
O1—S1—C16	108.48 (10)	C11—C12—C13	120.0 (3)
O2—S1—C16	108.97 (9)	C11—C12—H12	120.0
N2—S1—C16	106.60 (8)	C13—C12—H12	120.0
C15—N1—N2	115.26 (15)	C8—C13—C12	119.4 (3)
N1—N2—S1	113.54 (12)	C8—C13—H13	120.3
N1—N2—H1N2	115.6 (15)	C12—C13—H13	120.3
S1—N2—H1N2	113.8 (15)	C7—C14—C15	125.42 (17)
C6—C1—C2	120.84 (19)	C7—C14—H14	117.3
C6—C1—H1	119.6	C15—C14—H14	117.3
C2—C1—H1	119.6	N1—C15—C14	118.83 (16)
C3—C2—C1	120.5 (2)	N1—C15—H15	120.6
C3—C2—H2	119.8	C14—C15—H15	120.6
C1—C2—H2	119.8	C21—C16—C17	120.0 (2)
C2—C3—C4	119.55 (18)	C21—C16—S1	119.57 (16)
C2—C3—H3	120.2	C17—C16—S1	120.44 (18)
C4—C3—H3	120.2	C16—C17—C18	118.8 (3)
C3—C4—C5	120.34 (19)	C16—C17—H17	120.6
C3—C4—H4	119.8	C18—C17—H17	120.6
C5—C4—H4	119.8	C19—C18—C17	121.9 (3)
C4—C5—C6	121.15 (18)	C19—C18—H18	119.0
C4—C5—H5	119.4	C17—C18—H18	119.0
C6—C5—H5	119.4	C18—C19—C20	118.3 (2)
C1—C6—C5	117.65 (16)	C18—C19—C22	120.6 (3)
C1—C6—C7	119.93 (16)	C20—C19—C22	121.1 (3)
C5—C6—C7	122.42 (16)	C19—C22—H22A	109.5
C14—C7—C6	121.50 (16)	C19—C22—H22B	109.5
C14—C7—C8	121.27 (15)	H22A—C22—H22B	109.5
C6—C7—C8	117.23 (14)	C19—C22—H22C	109.5
C13—C8—C9	118.7 (2)	H22A—C22—H22C	109.5
C13—C8—C7	121.79 (19)	H22B—C22—H22C	109.5
C9—C8—C7	119.53 (17)	C19—C20—C21	121.3 (3)
C10—C9—C8	121.7 (2)	C19—C20—H20	119.3
C10—C9—H9	119.2	C21—C20—H20	119.3
C8—C9—H9	119.2	C16—C21—C20	119.7 (2)
C11—C10—C9	119.4 (3)	C16—C21—H21	120.1
C11—C10—H10	120.3	C20—C21—H21	120.1
C9—C10—H10	120.3		

### Hydrogen-bond geometry (Å, °)

**Table:**

*D*—H···*A*	*D*—H	H···*A*	*D*···*A*	*D*—H···*A*
N2—H1N2···O2^i^	0.82 (2)	2.11 (2)	2.927 (2)	173 (2)

Symmetry codes: (i) −*x*+1/2, *y*−1/2, −*z*+1/2.
